# Epidemic Erysipelas, as It Occurred in La Porte County, Indiana

**Published:** 1844-06

**Authors:** D. Meeker

**Affiliations:** La Porte, Ia.


### *Epidemic Erysipelas, as it occurred in La Porte County, Indiana.*

By D. Meeker, M. D., of La Porte, Ia.

From brief notices, from different parts of the country, it appears that an epidemic erysipelas has been prevailing quite extensively in many of the Eastern as well as the Western States, for two or three years; assuming various names in different parts of the country, as "black tongue," "spotted fever," &c. It is not my intention in this article to bring before the reader the pathology, symptoms, and treatment of this disease, as found in books of surgery or practice, but to confine my remarks to the history, symptoms, treatment, and progress of the disease, as it occurred in La Porte County, Ia. It made its appearance at Michigan City, La Porte County, in December last, where it still prevails, although its violence has very much abated; from this place it spread into other parts of the County, and into the adjoining County, St. Joseph, assuming rather a milder form. The mortality of the disease at Michigan City has been great, out of about 60 cases, about 30 proved fatal. (Though not greater than in many other places as in some where this epidemic has prevailed, nearly every case was fatal.) The commencement of the disease was generally characterised by lassitude, cold or chilly sensations; in severe attacks, the cold stage lasting for some time, followed by high arterial action. In many cases, some swelling of the tonsillary glands, with an efflorescent or inflamed appearance of the mucous membrane of the fauces existed, with a dryness of the tongue, which was covered with a dark brown coat, neuralgic pains in the situation of the lymphatic glands of the neck, axilla, and frequently of the lower extremities. The inflammation did not make its appearance upon the skin in many cases, until the second or third day, and sometimes not until a later period. In other cases, the internal organs became inflamed, without its making its appearance upon the surface at all.

It seemed to attack the mucous membranes with avidity, extending sometimes from the inflamed fauces to the mucous membrane of the stomach, causing a burning sensation in that region, vomiting, great tenderness upon pressure over the epigastric region, and in many other cases, the parenchymatous structure of the lungs was the seat of the disease. The pain in the chest was not acute but dull, with difficult respiration, and short dry cough, attended with constriction. In three cases, the mucous membrane of the ileum and colon was the original seat of the disease. In these cases the pulse was hard, wiry and contracted, with great distension of the bowels, and pain and tenderness upon pressure. The inflammation frequently made its appearance upon the ala of the nose, spreading rapidly over the face and scalp, attended with great swelling and distention, distortion of the countenance, and the eyelids entirely closed. Vesications, filled with a yellowish fluid, usually made their appearance upon the inflamed surface about the second or third day. The face and scalp was the most common seat of the disease, although in some cases the superior or inferior extremities became inflamed, attended with great effusion of serum into the cellular membrane. In some instances, the inflammation was of a phlegmonous character, sometimes superficially seated, when it was attended with less constitutional disturbance than the more deep seated variety. It seemed to spread with greatest rapidity in damp weather. It also attacked nearly all puerperal females, not more than one escaping in ten cases at Michigan City. They were invariably attacked with a chill after the termination of labor, some as soon as six hours, others as late as the second day.—There was great tenderness upon pressure over the region of the uterus, which organ could be distinctly felt through the parietes of the abdomen, very much enlarged. In some of these cases of puerperal erysipelas, the inflammation involved the vagina and external organs of generation. The symptoms were generally as follows:

General lassitude, rigors, neuralgic pains, soreness, and swelling of the tonsils, stiffness of the muscles of the part before inflammation made its appearance on the surface, a disposition to spread from point to point, increased arterial action, pulse hard and rather contracted, frequency from 100 to 120, and all cases attended with more or less bilious derangement. The most successful plan of treatment consisted in bleeding from the arm until a decided impression was made upon the system. This followed by a full dose of calomel, and if necessary, some saline laxatives, or ol. ricini was given to quicken the operation. This cathartic seldom failed to bring copious discharges of bilious matter from the bowels.

It was seldom necessary to repeat the bleeding, except when the lungs or some other of the internal organs were involved in the inflammation. Alteratives combined with diaphoretics—such as a combination of ipecac. and calomel—with gentle laxatives, after the force of the disease had been broken down by more active means, was the course usually pursued. The local applications found to be most beneficial, were the nitrate of silver and blisters. The blistering, no doubt, affords more prompt relief than any other application. By it we unload the cellular membrane of the effusion which has taken place, more effectually than by any other means. After vesication had taken place, the blistered surface was dressed with the warm water dressing, or some other calculated to promote the discharge of serum. The nitrate of silver, applied to the inflamed surface by penciling the part, or in strong solution, affords a prompt and effectual method of arresting the inflammation, which is sometimes so rapid as not to be arrested by any milder means. In such cases, it should be applied by dipping the stick of caustic in water, and applying freely over the inflamed surface, or by completely encircling it by running the pencil round its edges. I have used the iodine in one case, and it arrested the progress of the inflammation almost instantaneously. It may be used in the form of ointment, on a saturated tincture sufficiently strong to act upon the skin promptly; in short, all local applications should be sufficiently powerful to excite an inflammation in the sound skin and subcutaneous cellular membrane, before the disease reaches that point. We thereby exchange an inflammation which is prone to spread from point to point, until it invades the whole snrface, for one which is simple and healthy in its character, and perfectly under our control.

When the inflammation was more deeply seated, suppuration of the cellular membrane was the most usual termination. In such cases free incisions into the diseased part were necessary to give free exit to the matter, and some emollient poultice applied to promote the discharge. In cases attended with much oedema of the extremities, giving a doughy feel, incisions or punctures, as recommended by Mr. Liston, may be resorted to with beneficial results. In order to illustrate this subject more fully, I will give a few cases from recollection. Miss W., aged 13 years, of good constitution, was taken on Friday with chilly sensations, pulse small and hard, pain in the abdomen, tenderness upon pressure, with considerable febrile disturbance. Dr. C. Palmer, the family physician, was called in, and the patient bled and a dose of calomel given, which operated without giving much relief. On Saturday she was again bled, a large blister applied to the abdomen, and she was put upon alterative doses of calomel, ipecacuanha, and morphine. I was called on Sunday in consultation, found the tenderness of the abdomen somewhat diminished, pulse fuller than before last bleeding, bilious discharge from the bowels, and erysipelatous inflammation making its appearance upon the nates, spreading to the vulva. The inflamed surface upon the nates was covered with blisters, which arrested the spreading of the disease, a strong solution of the nitrate of silver to the vulva, powders containing 2 grs. calomel, $\frac{1}{4}$ gr. ipecac every 3 hours, and a sufficient quantity of morphine to control the bowels was given, or laxatives if necessary to quicken the operation. About the time the inflammation appeared upon the surface, the tenderness and distention of the abdomen entirely disappeared and the patient gradually recovered.

In a few days the father was taken nearly in the same manner as the daughter, with all the symptoms much aggravated, together with retching and vomiting. The same course of treatment was pursued as in the other case. As nearly as I recollect, the patient died on the tenth day. A postmortem examination exhibited the following appearance. Mucous membrane of the ileum and colon in a high state of erysipelatous inflammation approaching to gangrene, the peritoneum not being involved in the disease. I would here remark, that so far as my observation extends, I have not seen any cases where the serous membranes were involved in this disease.

